# From optimistic to overwhelmed: exploring health beliefs and barriers to oral anticancer medication adherence among cancer survivors

**DOI:** 10.1007/s00520-026-11106-x

**Published:** 2026-08-21

**Authors:** Meng-Jung Wen, Maite A. Zapata, Olayinka O. Shiyanbola

**Affiliations:** 1 Division of Social and Administrative Sciences, School of Pharmacy, University of Wisconsin-Madison, Madison, WI USA; 2 School of Pharmacy, University of Wisconsin-Madison, Madison, WI USA; 3 Department of Clinical Pharmacy, College of Pharmacy, University of Michigan, Ann Arbor, MI USA; 4 Department of Pharmaceutical Sciences, Texas A&M University Irma Lerma Rangel College of Pharmacy, College Station, TX USA

**Keywords:** Health beliefs, Medication adherence, Cancer survivorship, Oral anticancer medications, Patient-centered care, Qualitative research

## Abstract

**Purpose:**

This study explored cancer survivors’ health beliefs about oral anticancer medications (OAMs) and examined how these beliefs influence adherence-related decision-making, while identifying barriers and strategies to support cancer management.

**Methods:**

Twelve patients with cancer using OAMs, previously categorized into three health belief profiles, were interviewed virtually. Thematic analysis and theme mapping were used to examine the complexity of how survivors formed beliefs about cancer and OAM use.

**Results:**

Survivors expressed diverse beliefs that shaped coping, adherence, and engagement with care. Three domains emerged: positive beliefs reinforcing trust in providers and treatment; negative beliefs focused on recurrence, side effects, and systemic barriers; and varied beliefs about OAMs influenced by information sources and support networks.

**Conclusions:**

Survivors’ health beliefs influenced adherence and coping, underscoring the need for tailored education, communication, and support. Personalized, profile-based interventions are essential to strengthen treatment confidence and guide patient-centered survivorship care.

**Supplementary Information:**

The online version contains supplementary material available at https://doi.org/10.1007/s00520-026-11106-x.

## Background

Cancer is a leading cause of death in the United States, with an estimated prevalence of 5.5% [1]. In 2022, approximately 18 million people were diagnosed with cancer in the U.S., and this number is anticipated to rise to 22.2 million by 2030 [2, 3]. The healthcare system allocates nearly US$208.9 billion annually to address this pressing issue and the cancer-related out-of-pocket costs are estimated at 16.2 billion from patients [2]. In this study, the term ‘cancer survivors’ refers to individuals living with and beyond a cancer diagnosis, including those currently undergoing treatment. Nonadherence to cancer therapies significantly jeopardizes patient outcomes, with nearly half of cancer survivors failing to adhere to prescribed oral anticancer medications (OAMs). This nonadherence not only adversely affects the patients but also imposes burdens on their families and society at large. Recent studies reveal that adherence rates for certain cancer types can be as low as 33%, far below the general adherence rate of 50% observed in chronic diseases [4]. Nonadherence is associated with serious consequences, including 20% of early cancer recurrence, four times of hospitalization rates, and premature mortality [5–7]. Moreover, it exacerbates financial hardships, as nonadherent patients face 2.8 times out-of-pocket expenses compared to those who adhere to their OAM regimens [2, 8–10].

Empirical studies increasingly indicate that negative patient beliefs have resulted in nonadherence to cancer treatment, presenting a major obstacle to effective cancer management. Common barriers to adherence to OAMs include side effects, limited disease-related knowledge, forgetfulness, reduced perceived treatment efficacy, and financial burden [7, 11, 12]. Although cancer type, stage, prognosis, treatment pathway, and medication side effects are important contextual influences, systematic reviews suggest that individual-level psychosocial barriers, particularly illness perceptions, beliefs about medicines, emotional responses, and patients’ understanding of treatment, may play a more proximal role in shaping adherence-related decisions than demographic or clinical characteristics alone [12, 13]. This argument is further supported by our prior quantitative findings, which showed that distinct health belief profiles were significantly associated with medication adherence and health-related quality of life [14].

The Extended Common-Sense Model of Self-Regulation indicates that preconceived notions about illness, including beliefs about medication, play a critical role in the decision-making process for medication use among patients with chronic disease [15–19]. Among cancer survivors, this process often involves balancing positive perceptions (e.g., the need for longer survival and symptom reduction) against negative perceptions (e.g., concerns about side effects and uncertainty) regarding taking OAMs [18, 20–22]. When weighing the benefits and risks of taking OAMs, survivors usually engage in a deliberative process, making compromises to arrive at a final decision – whether to adhere to the treatment or modify the regimen as a mean of regaining control of their disease [23, 24]. This decision-making process is particularly evident in individuals with advanced stages of cancer, where the uncertainty surrounding medication efficacy is weighed against the cumulative burdens experienced over time [23, 24]. Given the complexity of how positive and negative perceptions interact in these decisions, it is essential that interventions targeting nonadherence incorporate personalized strategies to address specific barriers effectively.

To explore diverse patterns of health beliefs, adopting person-centered approaches to cluster potential users into subgroups offers an innovative and alternative strategy for addressing patient-level barriers to medication adherence. Increasingly, personas are being utilized in the development of innovative health services and interventions by identifying patients’ needs, goals and challenges [25–27]. The process of developing personas for health interventions not only uncovers individual thought patterns but also delves deeper into patients’ experiences, including their daily routines, decision-making preferences, core values in disease management, and the need for support systems. However, few studies have thoroughly examined how individuals form their perceptions and how these perceptions influence their decision-making processes [27]. Furthermore, it is critical to identify and address patients' supportive needs for disease management while aligning these needs with their health belief patterns when designing targeted interventions. Therefore, the primary objective of this study was to explore cancer survivors’ health beliefs and examine how these beliefs influence the decision-making processes regarding the use of OAMs. Additionally, this study aimed to identify individual barriers and potential strategies to enhance medication adherence and improve overall cancer management.

## Methods

### Study design and procedure

This qualitative study utilized stratified sampling to recruit participants based on the three health belief subgroups/profiles from the previous quantitative phase. In the quantitative phase, participants were recruited across the United States through two online crowdsourcing platforms, Centiment® and Amazon Mechanical Turk®. Participants in the parent survey were eligible if they were 18 years or older, self-reported a cancer diagnosis, were currently taking at least one OAM, and were able to read and write in English. Participants were excluded if they did not consent or failed survey quality checks. Eligible participants completed an online survey through Qualtrics and were subsequently categorized into three health belief profiles using latent profile analysis – *Optimistic and adaptive*, *Conscious but hesitant*, and *Overwhelmed and unaware* group, among cancer survivors taking OAMs. These subgroups were characterized by differences in beliefs about cancer and OAM use, providing the foundation for qualitative sampling to explore survivors’ lived experiences in greater depth. Additionally, purposive sampling was conducted to ensure diversity in age, cancer type, cancer stage, and treatment duration, within each subgroup, allowing for a broader range of experiences to be captured. Participants were eligible for this qualitative study if they (1) completed the survey and provided their contact information at the end of the survey and (2) agreed to participate in the interview. This study was conducted between August and October 2024 in accordance with the ethical standards of the Declaration of Helsinki. Ethical approval was obtained from the University of Wisconsin–Madison Institutional Review Board (IRB) (Protocol ID: 2024–0549). Informed consent was obtained from all individual participants included in the study. Confidentiality was maintained through data de-identification, encrypted storage, and restricted access. Clinical trial number: not applicable.

### Data collection

A semi-structured interview guide was developed based on the domains of participants’ health beliefs and questions related to medication adherence within the Extended Common-Sense Model of Self-Regulation to capture participants’ perceptions [15, 18]. Based on the ANOVA results comparing significant subgroup differences in health beliefs and the multiple liner regression results of significant predictors of nonadherence, the final set of interview questions was selected using two criteria – f*irst*, health belief domains with significant subgroup differences (*p* < 0.05) among the three health belief subgroups, including nine domains – Cancer Consequences, Risk of Recurrence, Emotional Representations, Coherence, Personal Control, Treatment Control, Treatment Consequences, Necessity and Concerns, and *second,* health belief domains identified as strong predictors (*p* < 0.05) of nonadherence in the subgroup with lowest adherent (*Profile 3: Overwhelmed and Unaware group*). Two additional domains, including health literacy and comorbidities, were also added to the interview guide to explore their role in shaping participants’ experiences and to understand how and why these factors emerge as barriers to taking OAMs. Interview questions related to health literacy focused on participant experiences in understanding and utilizing health information throughout their cancer journey. Supplementary Information 1 presents the questions used in the semi-structured interviews.

The initial draft of the interview guide was revised by experts with extensive experience in qualitative research and patient perceptions. Pilot testing was conducted with the first two participants, and further revisions were made based on feedback from experts and participants. The interviews were conducted by the researcher, a registered pharmacist with six years of intensive qualitative methodological training and experience in conducting qualitative studies during graduate school. In-depth, one-on-one virtual interviews were conducted via Zoom, with each interview lasting between 20–60 min. Recruitment continued until thematic saturation was reached, where no new concepts emerged from interviews [28]. All online interviews were audio-recorded, and the interview data were securely stored in an encrypted drive in the researcher’s office. The study adhered to the Consolidated Criteria for Reporting Qualitative Research (COREQ) checklist to ensure methodological rigor, transparency, and credibility in study design, data collection, and analysis [29].

### Data analysis

Thematic analysis, guided by Braun et al., was used to explore (1) cancer survivors’ beliefs about cancer and oral anticancer medications (OAMs), (2) how their health beliefs influenced medication-taking behaviors, and (3) barriers to taking OAMs as well as strategies to support adherence for future interventions [30]. All interviews were transcribed verbatim, with personal information de-identified. The first author and a researcher with a qualitative research background conducted deductive coding of the transcripts based on predefined domains in the theoretical framework. Additionally, inductive coding was applied to identify themes not covered by the framework.

The analysis proceeded iteratively, beginning with a comprehensive understanding of the transcripts through line-by-line reading and the extraction of initial codes. Subsequently, codes for each domain were compiled to identify overarching themes addressing the main research questions. In the third phase, the extracted themes were reviewed to ensure they covered all codes and to identify any overlaps. A categorization matrix and theme mapping were employed to visualize each domain of beliefs about cancer and OAMs across the health belief subgroups identified in the quantitative phase. Finally, each theme was labeled with a specific name and defined clearly [30].

To ensure the trustworthiness of the qualitative data in this study, four principles—credibility, transferability, dependability, and confirmability—guided by Lincoln and Guba were applied [31]. To establish credibility, researchers immersed themselves in the data, reading the entire transcripts for a general understanding of participants’ beliefs and conducting line-by-line coding to capture meaningful units of experience. Two independent coding of all transcripts were conducted to achieve investigator triangulation. Dependability was ensured through peer debriefing with experts who have experience with qualitative studies. This process involved confirming that the codes, themes, and interpretations aligned with the research questions during regular meetings with an expert who was not involved in the research process. Thick descriptions of the procedures, data collection, and analysis were provided in the final results to ensure transferability. Comprehensive data management was used to track all coding and theme trails using MAXQDA Version 2020. Any changes regarding research questions or procedures for data collection and analysis were documented with rationales to establish confirmability.

## Results

### Participant demographics

A total of 12 participants agreed to participate and completed the virtual interviews. The average age of participants was 64.0 years (SD = 11.2). Among them, three were male (3/12, 25%), and the majority (9/12, 75%) were White. Almost all participants (11/12) attended college or higher education and lived in urban or suburban areas. The average interview duration was 50.5 min (range from 15 to 96 min). Five out of twelve (41.7%) participants were diagnosed with breast cancer, with an average duration since diagnosis of six years (range from three months to 26 years). Half of participants were in their early stages of cancer (stage 0–2), and five had experienced cancer recurrence. Most participants were taking oral hormone therapy, with nine of them (75%) having been on OAMs for more than one year. Table 1 lists participant demographic information.

**Table 1:** Participant demographic information

No	Profile^*^	Gender	Age	Race	Education	Employment	Urbanicity	Cancer type	Cancer duration	Cancer stage	Recurrence	Type of OAMs	OAM duration	Comorbidity
1	3	F	35	Black	Below college	Unemployed	Urban	Endometrial cancer	12	3	Yes	Targeted therapy	> 5 years	0
2	3	F	61	White	Degree or above	Full time	Urban	Breast cancer	3	3	No	Hormone therapy	3–5 years	2
3	1	M	72	White	Degree or above	Retired	Urban	Colon and rectal cancer	4	3	No	Don't know	1–3 years	3
4	2	F	69	White	Degree or above	Part time	Suburban	Breast cancer	0	1	No	Hormone therapy	< 3 months	0
5	3	F	67	White	Degree or above	Part time	Urban	Thyroid cancer	2	N/A	Yes	Hormone therapy	1–3 years	2
6	2	F	72	White	Degree or above	Full time	Urban	Breast cancer	26	N/A	Yes	Hormone therapy	3–5 years	1
7	1	F	68	White	Degree or above	Retired	Urban	Ovarian cancer	10	1	Yes	Hormone therapy	> 5 years	2
8	1	F	78	White	Degree or above	Retired	Suburban	Breast cancer	2	2	No	Hormone therapy	1–3 years	5
9	2	F	59	White	Degree or above	Full time	Suburban	Breast cancer	9	2	No	Hormone therapy	3–5 years	0
10	3	M	55	White	Degree or above	Disabled	Suburban	Colorectal cancer	2	2	No	Targeted therapy	3 months–1 year	5
11	2	M	70	Hispanic white	Degree or above	Unemployed	Suburban	Prostate cancer	1	2	No	Don't know	1–3 years	5
12	2	F	62	Asian	Degree or above	Full time	Urban	Lung cancer	1	4	No	Targeted therapy	3 months–1 year	0

^*^Profiles were derived from latent profile analysis among cancer survivors taking OAMs: Profile 1, optimistic and adaptive; Profile 2, conscious but hesitant; and Profile 3, Overwhelmed and unaware

### Overarching findings of health beliefs among cancer survivors

**Fig. 1:**
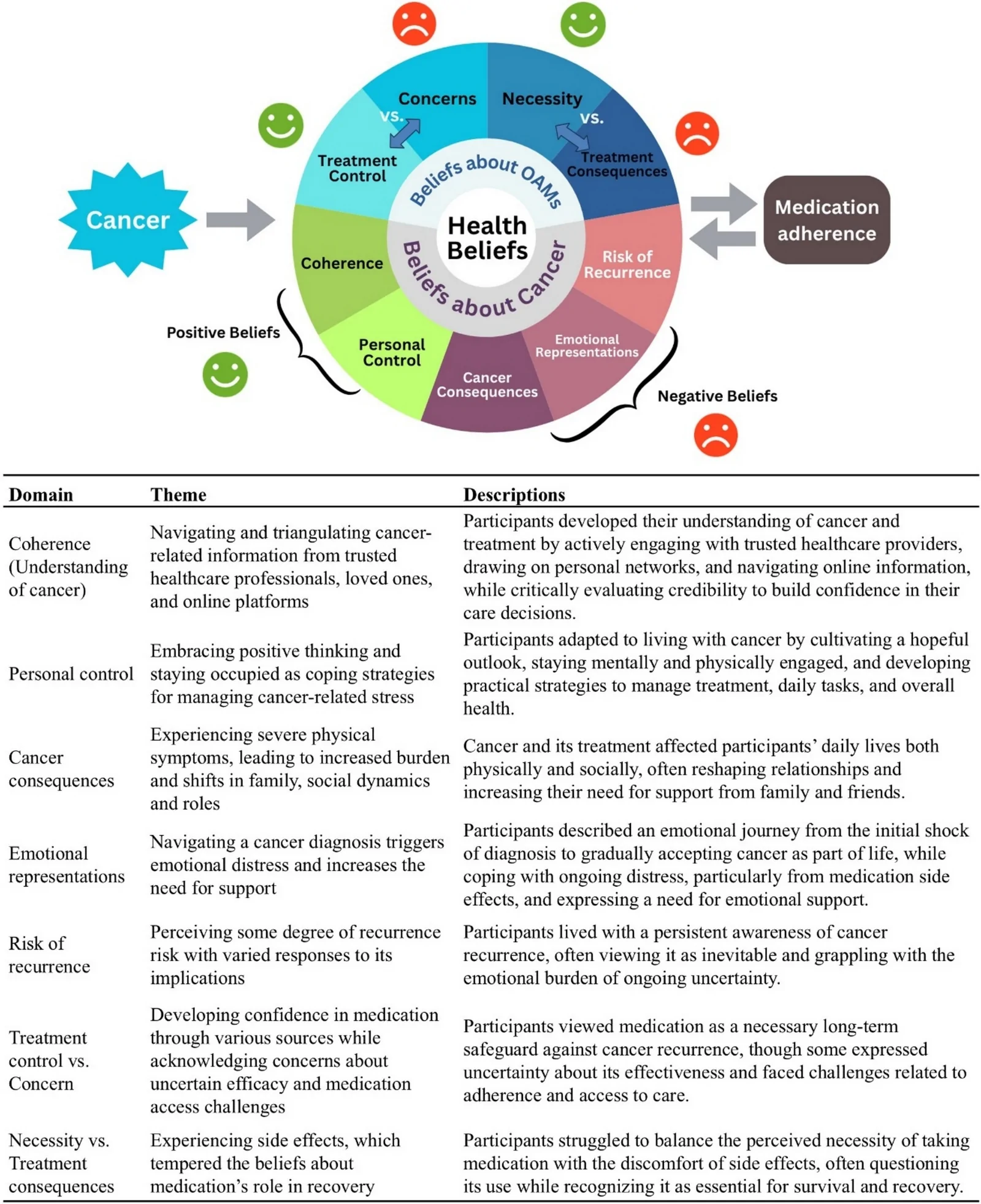
Mapping themes to health belief domains guided by the common sense model of self-regulation along with a summary of each theme’s findings

Guided by the Leventhal’s Common-Sense Model of Self-Regulation, findings related to patients' beliefs about cancer and oral anticancer medications were coded into three categories: (a) positive beliefs about cancer, including *Coherence* (understanding of cancer) and *Personal control* (b) negative beliefs about cancer, including *Cancer consequences*, *Risk of recurrence*, *Emotional representations* and (c) beliefs about oral anticancer medications, including *Treatment control*, *Necessity*, *Treatment consequences* and *Concern*. Figure 1 illustrated how the themes align with health belief domains and provides a summary of each theme’s findings. The theme, subtheme, codes and supportive quotes for each category are presented in Tables 2, 3, and 4.

**Table 2:** Theme, subtheme, code, selected subcode and quotes for category 1: positive beliefs about cancer

Subtheme	Code	Selected subcode and quote
Theme 1: Navigating and triangulating cancer-related information from trusted professionals, loved ones and online platforms (Coherence, understanding of cancer)		
Developing cancer understanding and trust in cancer treatment through healthcare providers	Trust in healthcare providers and the system	“What’s the treatment plan going to be? And I trusted in my doctors. and they’re capabilities to get me through the process.” *Participant 3, Profile 1: Optimistic and adaptive group*
	HCPs’ role in explaining medication-related information	*Inconsistencies in cancer information* “The pharmacist, when he told me: It’s cancer medication, there is no substitute. and I walked out into my car and just started crying and thought, why didn’t anybody tell me?…” *Participant 5, Profile 3: Overwhelmed and unaware group* *Helped with understanding how cancer medication works in the body* “Some of it (the information about medication I’ve learned) is from my own reading, and some of it is what my doctors have told me.”* Participant 8, Optimistic and adaptive group*
	Strategies to help with understanding medical information	*Recognize patient’s emotional reactions and ensure readiness for learning cancer information* “They (Doctors) just they were really composed and calm, which was a really good thing, because it’s needed at a time like that … they told me they knew it was really hard news to hear, so give me time to compose myself to really they let it sink in first before continuing.” *Participant 1, Profile 3: Overwhelmed and unaware group* *Use plain language or graph to help with understanding* “With my oncologist there were a couple of times on visits where she had. She had printed out some materials for me to become better acquainted with stuff, and some of the materials would have drawings or charts. Or whatever, and she would at least briefly, you know, explain what she was showing me.” *Participant 3, Optimistic and adaptive group* *Take initiative to ask HCPs questions or clarify on jargons* “(If I have questions or confusion,) I just asked them. You know they were very good. Well, they were very good at explaining things as though I didn’t have a medical background. Because a lot of people don’t.”* Participant 7, Optimistic and adaptive group*
Reling on family and friends’ experience on cancer understanding	“My husband’s experiences played a very strong role into my feelings…I’ve lived with it for so long, except not personally in my body, but with my husband, with my mom, with my sister.” *Participant 4, Profile 2: Conscious but hesitant group*	
Navigating and evaluating online information for support and understanding	Questioning about the credibility of information online	“You can find information on Google about cancer which might be wrong. So that can hinder me from and anyone else from getting the right information.” *Participant 1, Profile 3: Overwhelmed and unaware group*
Theme 2: Embracing positive thinking and staying occupied as coping strategies for managing cancer-related stress (Personal control)		
Subtheme	Code	Selected subcode and quote
Shifting perspectives on cancer	Positive thinking	“I also think keeping busy in some way, whatever it is. … You don’t have to be great at whatever it is you’re doing but find some interest. Get out of the world of cancer.” *Participant 4, Profile 2: Conscious but hesitant group*
	Recognition of cancer as a manageable condition	“Don’t despair. There’s been so much progress in treatments and keeping you healthy and keeping you alive. … It’s not a death sentence. It’s just an illness. Put it in perspective.” *Participant 4, Profile 2: Conscious but hesitant group*
	Hope in modern cancer care	“It (Conquering cancer) is a work in progress… As my surgeon said, you know, 25 years ago we didn’t have a whole lot of options. But look at all the progress that we’ve made in the last 25 years.” *Participant 8, Optimistic and adaptive group*
Using distraction and keeping occupied as coping strategies	“I also think keeping busy in some way, whatever it is, you know. Reading, write, keep a journal, put your thoughts down. Paint, take a painting, you know who cares what it looks like. You don’t have to be perfect. You don’t have to be great at whatever it is you’re doing but find some interest. Get out of your, the world of cancer.” *Participant 4, Conscious but hesitant group*	
Developed strategies to manage tasks associated with taking medication and overall disease management	Setting reminders and using tools	“I set up an alarm on my cell phone… When I need to take medication, and then I have a sheet. I just write down what time I took the medication.” *– Participant 12, Profile 2: Conscious but hesitant group*
	Engagement in proactive health behaviors	*Maintaining a healthy lifestyle* “I think it’s very important to have like a healthy lifestyle. eat healthy food. Like More vegetables like a healthy diet and the system. I think it’s a very important part. you know, prevent we cannot prevent. You don’t know if you can prevent by eating by, you know. But it’s you know. It’s good chance.”* Participant 2, Overwhelmed and unaware group*
	Coordinated management of cancer and comorbidity	“I take a lot of medications because I’m very overweight. I have arthritis. I have high blood pressure…. The cancer pill and the cancer itself are probably the least. … that would affect least. I just every once a month I do all my pills in a little box, and I make it easy for my Statin, and you know I don’t feel burden from the cancer at all.”* Participant 7, Optimistic and adaptive group*
	Vigilance in cancer monitoring	“I am pretty closely watched. I do blood work. I do scans. I did have a scare, and it ended up being nothing. But it’s very scary when that the scare happened. I just. I try to be very vigilant. I’m very good about doctors appointments if they say you need to do blood work every so often. I do it every so often. I’m very good at following the orders for that, because I think that’s the way to keep me alive is to watch it.” *Participant 9, Conscious but hesitant group*

**Table 3:** Theme, subtheme, code, selected subcode and quotes for category 2: negative beliefs about cancer

Subtheme	Code	Selected subcode and quote
Theme 1: Experiencing severe physical symptoms, leading to increased burden and shifts in family, social dynamics and roles (Cancer consequences)		
Impact of cancer and treatment on daily life	Physical effects	*Cancer effects* “… I lost a couple of pounds in 2 weeks I was down to 94 …I could be out shopping and going to lunch, or something I might say, hey, I gotta go home. I want to go to bed.” *Participant 5, Profile 3: Overwhelmed and unaware group*
	Social effects	*Changes in body image and social identity* “(People would think) it’s important to have breasts if I go out and buy these artificial things to put on to make me look like I have breasts, you know. I’m still me, and I think that’s the message that I’m like giving myself. I’m not lesser because I had my breasts removed….I see them kind of trying not to look, but noticing that I’m different, and that hurts.” *Participant 4, Profile 2: Conscious but hesitant group* *Social isolation* “I don’t say pity parties I have to go through like, oh, you get better, or it’s I will find we’ll help you. We’re here for you. It’s like people treat me like I’m very fragile. being, which is really uncomfortable at times. So yes, I tend to avoid people. …But I have withdrawn from society a lot.” *– Participant 1, Overwhelmed and unaware group* *Reduced work hours and disruption* “…I am not working. I would like to work and I want to work, and we need to work unless. I need to be able to keep it that way, and I need to be able to earn money.” *Participant 11, Conscious but hesitant* *Financial effects* “It got to a point where it couldn’t cover for the medication, which is really expensive. I had to use my college fund for that. Which means I haven’t attended college up to now.” *Participant 1, Profile 3: Overwhelmed and unaware group*
Changed the dynamics with friends and family	Strengthened relationship with loved ones	“(During the doctor’s visit) my daughter was on the phone, and my daughter lives out of state so she couldn’t be there. But she wanted to be there and insistent, mom, you have to have her let me listen to the conversation. So for that whole hour we just had a good back and forth, and I felt very comfortable going into surgery.” *Participant 8, Optimistic and adaptive group*
	Needs for support from family and friends	“I don’t really know if I know what’s the best support I need, because pretty much I’m by myself in the United States. So, even though I have some friends. I have really good friends that they willing to support me, but I knew This kind of chemotherapy process is a long process, not just one day.” *Participant 12, Conscious but hesitant*
Theme 2: Navigating a cancer diagnosis triggers emotional distress and increases the need for support (Emotional representations)		
Subtheme	Code	Selected subcode and quote
Transition from shock to active coping	Trama of hearing “You Have Cancer”	“The way he (doctor) talked to me it’s like… you don’t have too long to live, anyway. Just enjoy your life… I was kind of shock, but I have no emotion at that point during the appointment.” *Participant 12, Profile 2: Conscious but hesitant group*
	Confusion and frustration from delayed cancer information	“I still didn’t know it was cancer medication. (…Until) I talked to the pharmacist, …I had no idea anything was that serious and basically went out to the car and cried.” *Participant 5, Profile 3: Overwhelmed and unaware group*
	Avoidance and fear of reality	“(My doctor) expecting me to go home and become as familiar with material as I felt like doing, and sometimes I didn’t. Yeah, I guess there was a little part of me. It felt like if I really dug deeply into this. that I was gonna see some darkness.” Participant 3, Optimistic and adaptive group *Feeling worried about the lack of available treatment options* “I got a sense of panic. And worry, because, having something like cancer means, you might get well, you might not. So hearing that the medicine isn’t working for you anymore is really tough. So I did panic at the time.”* Participant 1, Overwhelmed and unaware group*
	Acceptance of cancer as a long-term condition in life	*Motivation to cope for family’s sake* “I just had to accept it. I had to… for the sake of my family. for the sake of my husband, who I had just married a few months before that (having cancer diagnosis) and I was like, and I didn’t want to let him down.” *Participant 6, Conscious but hesitant group* *Cancer as a long-term companion* “I would describe it as a neighbor who never bothers me. Go never causes me any trouble…And I know they're there, but they never talk to me. You know they’re not unfriendly, but they’re just there.” *Participant 7, Optimistic and adaptive group*
	Living with gratitude as a survivor	*Shifting from receiving to giving* “I just feel very lucky, because so many people don’t have the outcome that I have…. when I am healed, which I really am, I want to be one of the people who do the helping.” Participant 4, Profile 2: Conscious but hesitant group
Emotional distress linked to medication side effects	Accepted side effects as an unavoidable part of treatment	“Sometimes I’ll get a little bit depressed, but I just think, in the back of my mind, you know what, this is a side effect…Take it as a grain of salt. You know if you need go, lay down and take a nap, when you get up you’ll feel better, and that’s a lot of times what I’ll do.”* Participant 9, Conscious but hesitant group*
	Needs for emotional support	“I think that’s a big part of how you get through cancer is by allowing yourself to be supported, you know, not saying I don’t. I don’t need any help. That’s that would be a terrible decision. Because, you’re building your own wall if you do that and it’s not realistic, because we all need help at times.” *Participant 4, Conscious but hesitant group* *Received support from family and friends* “I was very worried. And I think this is where and I was toxic, and my friends all said that maybe I should start taking…some anti-anxiety medication, because I just was desperate, and I just had a lot of anxiety. …I had a lot of friends around the country, and they called me all the time so I could talk to someone every day if I needed to. I was never really alone.”* Participant 7, Optimistic and adaptive group* *Emotional support from cancer support group* “It’s so helpful for me to participate (cancer) support group…And the good things I can do are not necessary to go in person. I can go online…people exchange information about treatment.”* Participant 2, Profile 3:Overwhelmed and unaware group*
Theme 3: Perceiving some degree of recurrence risk with varied responses to its implications (Risk of recurrence)		
Code	Selected subcode and quote	
Perception of inevitability of recurrence	“I guess it’s in their gene or in their DNA. … But I’d say that’s one thing I see would make the cancer come back.” *Participant 1, Profile 3: Overwhelmed and unaware group* *No medications were available at that time and cancer reoccurred* “There were no further drugs in those days for that particular cancer, and was like a triple negative thing. So after 5 years of going to the office first it was every few months, and then it was, you know, finally got to. It was yearly. and they said, you're fine, you're great. I thought I would never get it again. and I got it (cancer recurrence) 21 years later.” *– Participant 6, Conscious but hesitant, with the history of cancer recurrence*	
Facing the risk of cancer recurrence	*Confidence in facing recurrence* “(Cancer) could hit again at any point. …you’ve got moral support from Caring Bridge [app for sharing illness stories with close friends] and all your family. …*I’ll deal with it again if it does*.” *Participant 3, Profile 1: Optimistic and adaptive* *Feeling worried about recurrence* “My biggest problem with keeping myself cancer cancer-free or in remission. I’m sure I don’t want to hear here that get the diagnosis. I have cancer somewhere else, because that can happen right?” *Participant 11, Conscious but hesitant group*

**Table 4:** Theme, subtheme, code, selected subcode and quotes for category 3: beliefs about oral anticancer medications

Subtheme		Code	Selected subcode and quote
Theme 1: Developing confidence in medication through various sources while acknowledging concerns about uncertain efficacy and medication access challenges (Treatment control vs. Concern)			
Medication as a long-term safeguard		Taking medication can prevent cancer from coming back	“I think it (medication) is helping me. …So the chance of that type of cancer coming back, I’m sure, would be minimum because of me doing the hormone blocker.”* Participant 9, Conscious but hesitant group*
		Cancer is manageable with medication	“The last time I went to the oncologist they told me that the studies now are showing that with my form of ovarian cancer, Letrozole has been helping other people as well, and that they are now thinking that people like me wouldn’t even have to go through chemotherapy anymore because it doesn’t help. So you know, I was real happy to hear that that when I first started taking it 6 years ago. They didn’t really know that much about how it would help me.”* Participant 7, Optimistic and adaptive group*
		Relied on external sources of information to develop beliefs that medication can help	*Learned information from cancer support group* “We had talks about the cancer in general. How to manage it. What kinds of food you should avoid or you should take, and physical activities. You could do for (as) a cancer patient.” *– Participant 2, Overwhelmed and unaware group* *Learned cancer information online* “Usually (information) it’s on Internet and Google. Also, talking is a doctor, who is a nutritionist also helpful to understand. What’s kind of fruit. What kind of fruit and vegetables? It’s good to prevent cancer (I) could ask them.. Sometimes I go to Google, … and check from there.” *Participant 2, Overwhelmed and unaware group*
Lack of confidence in adhering to medication		Uncertainty about treatment effectiveness	*Doubt about medication’s true benefits* “My doctor is going to allow me to stay on it longer. Which is more formal than anything. But I want to know. Is that really going to help me? There’s not nothing bad’s gonna happen. But is it? Is there? Is there a positivity of staying on it longer?” *Participant 9, Conscious but hesitant group* *Medication offers some level of protection without guarantees* “No, no promises, I mean, no promises are made, but just what a great protection that this will be! So it was all in a positive light.”* Participant 4, Conscious but hesitant group*
		Access to medication or healthcare services	“Sometimes it’s really hard to get to the physical treatment sessions, the ones where you have to go to the clinic. So sometimes I’d wake up, I’d be really weak. And that was a really big challenge, considering we live a bit far from the clinic.” *Participant 1, Overwhelmed and unaware group* *Not having problems in accessing medication* “My medication is generic. It’s tier one. I have no copay. So. No, I haven’t run out of medication.” *Participant 8, Optimistic and adaptive group*
Theme 2: Experiencing side effects, which tempered the beliefs about medication’s role in recovery (Necessity vs. Treatment consequences)			
Subtheme	Code		Selected subcode and quote
Balancing the necessity of taking medication with unflavored symptoms or side effects	“Now it’s because I know when I was on Tamoxifen there was a Tamoxifen Facebook support group, and everybody complained. And you know what. I’d rather be alive with a few with some minor with some side effects, than be 6 feet under.” *Participant 9, Conscious but hesitant group*		
	Questioning the necessity of taking medications		“I have always believed that medication is there to make you better? although I would say it doesn’t feel that way in this situation because I get a lot of symptoms, which make me uncomfortable like. I get nauseous like. I’m nauseous all the time I’ve learned to. I don’t remember what it feels like to not being nauseous.”* Participant 1, Overwhelmed and unaware group*
	Need to take the medications to live and recover		“I think it (medication) is a very important safeguard. I mean, it’s probably the most important part of my recovery.” Participant 4, Conscious but hesitant

### Category 1: Positive beliefs about cancer (understanding of cancer and personal control)

#### Theme 1: Navigating and triangulating cancer-related information from trusted healthcare professionals, loved ones, and online platforms (coherence, understanding of cancer)

Patients described navigating cancer-related information through a process of triangulation across healthcare providers, personal networks, and online resources, which together shaped their understanding of cancer and its treatment. Trust in healthcare professionals was central, particularly for those with *Optimistic and adaptive* beliefs, who reported greater confidence when complex medical information was explained in clear and respectful ways, reinforcing both comprehension and trust. In contrast, patients with *Overwhelmed and unaware* beliefs often struggled with inconsistent messages or difficulty understanding clinical explanations, which limited trust-building with providers. Alongside professional input, many patients, especially those in the *Conscious but hesitant* group, relied heavily on family and friends with cancer experience, whose personal stories offered comfort and practical insights but sometimes introduced biases that required cross-checking. Online platforms also provided information and peer support, but credibility varied. Patients with *Overwhelmed and unaware* beliefs remained vulnerable to misinformation, even when digital communities offered benefits.

#### Theme 2: Embracing positive thinking and staying occupied as coping strategies for managing cancer-related stress (personal control)

Patients emphasized the importance of cultivating resilience through positive thinking and structured daily routines as key strategies for coping with cancer-related stress and regaining a sense of personal control. Many described staying busy with hobbies, work, or social activities to distract from illness and maintain a sense of normalcy*. Optimistic and adaptive* and *Conscious but hesitant* survivors especially valued seeing cancer as a manageable condition rather than a death sentence. Hope in modern treatments and survival gains further reinforced this adaptive mindset, helping patients reduce feelings of helplessness. Alongside emotional coping, survivors also adopted practical strategies to manage medications and overall disease burden, such as using pill organizers, alarms, or apps to support adherence, coordinating multiple health needs, and maintaining health-promoting routines like exercise and diet. *Conscious but hesitant* patients stood out for their vigilance in monitoring symptoms, side effects, and lab results, which offered reassurance and preparedness for medical discussions.

### Category 2: Negative beliefs about cancer

#### Theme 1: Experiencing severe physical symptoms, leading to increased burden and shifts in family, social dynamics, and roles (cancer consequences)

Cancer and its treatment profoundly shaped patients’ daily lives, as physical symptoms such as fatigue, nausea, pain, and changes in appearance disrupted their ability to maintain routines, social connections, and work responsibilities. These challenges were particularly pronounced among patients with *Overwhelmed and unaware* beliefs, who described difficulties carrying out everyday activities, withdrawing socially, and facing financial strain due to treatment costs and reduced work capacity. Changes in body image and social identity further compounded distress, while reliance on family and friends became essential for both practical and emotional support. For some, this strengthened bonds and increased feelings of connectedness. For others, especially in the *Conscious but hesitant* and *Overwhelmed and unaware* groups, it caused strain, guilt, or feelings of being a burden when support was limited or relationships grew distant.

#### Theme 2: Navigating cancer diagnosis triggers emotional distress and increases the need for support (emotional representations)

A cancer diagnosis often initiated intense emotional distress, beginning with shock, fear, and avoidance, before gradually shifting toward acceptance and active coping as patients adjusted to their new reality. This transition varied across belief profiles: patients with *Optimistic and adaptive* views more readily reframed cancer as a manageable, long-term condition, while those with *Overwhelmed and unaware* beliefs often described confusion, fear, and difficulty processing inconsistent or delayed information. Emotional distress was further compounded by treatment side effects, such as fatigue, nausea, and hair loss, which disrupted daily life and reinforced feelings of vulnerability. Participants reported that coping with these challenges required sustained support, with family and friends offering encouragement and practical help, while cancer support groups provided a sense of solidarity and understanding.

#### Theme 3: Perceiving some degree of recurrence risk with varied responses to its implications (risk of recurrence)

Patients commonly acknowledged the possibility of cancer recurrence, but their responses ranged from resignation to proactive resilience. Those in the *Overwhelmed and unaware* group often viewed recurrence as inevitable and beyond their control, leading to more passive coping. In contrast, *Optimistic and adaptive* patients expressed confidence in their ability to face recurrence, drawing strength from prior experiences and medical advancements. For many, the risk of recurrence remained a persistent source of anxiety, especially during follow-up scans or when experiencing unexplained symptoms. Anxiety served to influence both their vigilance in health monitoring and their efforts to maintain a sense of normalcy.

### Category 3: Beliefs about oral anticancer medications

#### Theme 1: Developing confidence in medication through various sources while acknowledging concerns about uncertain medication efficacy and medication access challenges (treatment control vs. concern)

Patients described building confidence in their cancer medications through reassurance from healthcare providers, personal experiences, and external sources such as peer networks and online platforms. For many in the *Optimistic and adaptive* and *Conscious but hesitant* groups, medications were seen as a safeguard against recurrence, offering a sense of security and hope that cancer could be controlled with consistent treatment. In contrast, patients in the *Overwhelmed and unaware* group often relied more heavily on external information and the experiences of others to reinforce their trust in medication, highlighting a greater need for support in developing confidence. Despite this reliance, uncertainty about medication effectiveness persisted, as some participants doubted whether their treatment would truly protect them, viewing it as helpful but not guaranteed. Access barriers, whether financial, logistical, or tied to healthcare availability, further complicated adherence, particularly among those already struggling with limited trust or heightened vulnerability.

#### Theme 2: Experiencing side effects, which tempered beliefs about medication’s role in recovery (necessity vs. treatment consequences)

Patients described a conflicted relationship with oral anticancer medications, recognizing their necessity for survival while struggling with the burden of side effects. For some, persistent symptoms such as nausea or fatigue led to doubts about whether the benefits outweighed the discomfort, particularly among those in the *Overwhelmed and unaware* group. Despite these challenges, most patients acknowledged that discontinuing treatment was not an option, highlighting how adherence was maintained out of necessity rather than confidence, with side effects shaping both trust in medication and overall quality of life.

## Discussion

This qualitative study, guided by Leventhal’s Common-Sense Model of Self-Regulation, explored cancer survivors’ beliefs about cancer and OAM use, revealing a diverse range of perceptions that influenced their coping strategies, medication adherence, and engagement with healthcare. While some participants exhibited optimistic and adaptive beliefs, relying on healthcare providers, structured coping mechanisms, and modern treatment advancements, others demonstrated more hesitant or overwhelmed perspectives, shaped by uncertainties about recurrence, medication side effects, and systemic barriers to healthcare access. Variations in treatment confidence, information processing, and reliance on external support networks further distinguished participants’ experiences, emphasizing the need for personalized education, enhanced communication, and tailored support systems to improve survivors’ understanding and adherence to treatment.

Findings from this study illustrate the diverse sources of cancer-related information that patients utilize. While healthcare providers were often perceived as the most reliable and authoritative sources of information, reliance on informal networks and digital resources varied across patient subgroups. Survivors with *Overwhelmed and unaware*beliefs struggled to evaluate credibility, leaving them more vulnerable to misinformation and distrust. Their uncertainty often led them to rely on unverified online sources or personal anecdotes, increasing their vulnerability to misinformation and distrust in healthcare providers. These findings align with existing literature suggesting that cancer patients actively engage in information-seeking behaviors but may struggle with evaluating the credibility of online content [32]. Scoping reviews have also highlighted that while digital resources provide a wealth of health information, cancer patients frequently encounter challenges in distinguishing credible sources from misinformation [33]. Balancing the accessibility of digital health resources with safeguards against misinformation remains a key challenge. Healthcare providers should consider guiding patients toward reliable online sources and fostering open discussions about information credibility to support informed decision-making.

Beyond traditional medical consultations, family members and peer support networks played a crucial role in shaping patients' beliefs about cancer and treatment. Previous research highlights that patients with cancer often turn to personal networks for reassurance, emotional support, and practical advice [34, 35]. However, while firsthand experiences shared by family and friends can offer comfort, they may also introduce biases or misconceptions that influence treatment perceptions. This study found that patients in the *Conscious but hesitant* group relied heavily on familial advice, and cross-checking information with medical sources to mitigate potential misinformation. These findings underscore the need for structured educational and behavioral interventions that equip both patients and their support systems with accurate, comprehensible, and context-specific information about cancer management.

Structural barriers and social determinants of health continue to be critical challenges in cancer care, particularly for patients in the *Overwhelmed and unaware*group, who often face financial constraints, transportation difficulties, and healthcare access disparities. Research highlights that patients from lower socioeconomic backgrounds, uninsured individuals, and those in rural communities are disproportionately affected by these barriers, leading to delays in diagnosis and treatment, lower adherence rates, and poorer health outcomes [36]. Limited transportation access, for example, has been identified as a major obstacle, with many patients missing critical appointments due to limited access to reliable transportation services [37]. Financial toxicity is another pressing issue, as high out-of-pocket costs force some patients to delay or forego treatment, exacerbating disparities in survival rates [38]. These findings underscore the need for healthcare policies that address the structural inequities contributing to poor cancer outcomes. Programs such as patient navigation, financial assistance initiatives, and community-based health outreach efforts have demonstrated success in bridging gaps in care by improving access to critical resources and ensuring patients receive timely treatment [39]. Family-based interventions can also further support caregivers, helping to reduce patient burden and improve both adherence and emotional well-being [40–42]. Ultimately, a comprehensive, multi-level approach that integrates structural support with psychosocial care is essential to reducing disparities and improving patient outcomes [43]. Strengthening collaborations between healthcare providers, social services, and policymakers can help bridge these systemic gaps and ensure that the most vulnerable cancer patients receive equitable access to care.

Cancer survivors often weigh the necessity of OAMs against concerns about side effects, uncertain efficacy, and long-term risks. Findings from this study align with prior research that patients continually evaluate the trade-offs between treatment benefits and adverse effects, shaping their intention to take medications [23, 24]. To improve adherence and reframe medication beliefs, patient-provider communication strategies should be integrated into structured educational programs, ensuring that patients receive clear, individualized information about OAMs. Evidence suggests that leveraging digital health tools, such as mobile applications, online patient portals, and telehealth consultations, can facilitate real-time provider engagement and reinforce positive treatment perceptions [43–46]. For the *Optimistic and adaptive* group, digital reminders and educational resources can sustain confidence in their regimen. Survivors with *Conscious but hesitant* beliefs would benefit from interactive, evidence-based platforms that allow for ongoing dialogue and reassurance from providers. For the *Overwhelmed and unaware* group, a more intensive approach incorporating direct counseling, symptom-tracking applications, and caregiver involvement may help reduce concerns and build confidence in treatment necessity. Future interventions should explore hybrid models that combine in-person education with digital support, ensuring that all patients, regardless of their health literacy level, have access to reliable information and structured guidance for managing their treatment effectively.

### Study strengths and limitations

This study is among the first to explore how health beliefs influence cancer management, particularly treatment adherence by categorizing distinct health belief patterns. By adopting a subgroup approach, this study provides a nuanced understanding of how cancer survivors with different beliefs perceive their condition, experience challenges, and make decisions about their care. However, the small sample size within each profile may limit the generalizability of these findings. To address this, we leveraged the grouping nature of our study design to compare and synthesize insights across three distinct health belief patterns, maximizing our ability to identify variations in coping strategies and decision-making processes among cancer survivors. Despite these efforts, the study sample was predominantly White, female, and from urban or suburban areas with internet access. Given that health beliefs are shaped by social contexts and resource availability, findings should be interpreted with caution, as geographic and socioeconomic factors may influence individual perceptions. Although participants represented a range of cancer types and treatment experiences, this qualitative study was not designed to determine whether cancer type independently shaped participants’ beliefs or adherence-related experiences. Future research should examine whether individuals within the same belief profile may report different concerns, adherence barriers, or support needs depending on cancer type and treatment pathway. This would help determine whether belief-based tailoring should be further refined by cancer-specific treatment contexts. Furthermore, participants were generally more engaged in their health, potentially reflecting socially desirable responses. Some interviews were conducted with caregivers present, which may have influenced participants’ narratives [47]. To minimize the influence and enhance the quality of the responses, interviewers used probing techniques and rephrased questions to clarify responses and capture subjective experiences directly from participants.

## Conclusion

This qualitative study explored how health beliefs shape cancer management and medication-taking behaviors among cancer survivors, identifying distinct barriers, facilitators, and coping strategies across three health belief profiles. Findings revealed that survivors in the *Optimistic and adaptive* group exhibited strong self-efficacy, trust in healthcare providers, and proactive coping mechanisms, allowing them to navigate medication-taking with minimal concerns. The *Conscious but hesitant* group engaged critically with their treatment, requiring reassurance, structured support, and ongoing monitoring to balance concerns about treatment necessity and side effects. The *Overwhelmed and unaware* group faced significant challenges, including greater emotional distress, a higher perceived burden of cancer and treatment, and reliance on external sources for understanding their condition. Their limited sense of control and inconsistent information-seeking behaviors contributed to higher nonadherence risks. Beyond education, interventions should address emotional distress, enhance symptom management, and ensure access to reliable information and build treatment confidence. Future research should explore how personalized adherence strategies can be integrated into oncology care while considering the social, emotional, and structural factors that shape long-term adherence.

## Supplementary Information

Below is the link to the electronic supplementary material.

ESM 1 (DOCX 20.6 KB)

## Data Availability

The data that support the findings of this study are not publicly available due to restrictions related to participant confidentiality but are available from the corresponding author upon reasonable request.
