# Supplementary material for: From optimistic to overwhelmed: exploring health beliefs and barriers to oral anticancer medication adherence among cancer survivors
Source: Support Care Cancer. 2026 Aug 21;34(9):886. doi: 10.1007/s00520-026-11106-x (PMC13498498; doi:10.1007/s00520-026-11106-x)
Supplement: Supplementary file 1 [file 520_2026_11106_MOESM1_ESM.docx]

**Supplementary Information 1.** Semi-structured Interview Guide of Beliefs about Cancer, Beliefs about OAMs and Medication Adherence

| ***Section A: General experience*** | | |
| --- | --- | --- |
| **Topic** | **Semi-structured interview questions** | **Rationale or comment** |
| Profile name | 1. Could you briefly talk about your experience with cancer? 2. How would you describe your relationship with cancer? | Get input directly from participants of each profile. It may help with naming the profile. |
| Cancer medication/ Profile name | 3. What oral cancer medication are you currently taking? 4. What role do you think cancer medicines play in cancer? 5. What do you believe is the most important benefit of your current treatment? | This question is just to remind them that what we will be talking about during the interview is the specific medication(s) for treating their cancer |
| ***Section B: Beliefs about cancer*** | | |
| **Domains** | **Example interview questions** | **Rationale for questions** |
| Cancer consequences | 1. What do you see as the main effects of having cancer?    - How does (the main effect) influence whether you take your cancer medication? | - Understand the significant group differences in domains of beliefs about cancer among three health belief subgroups/profiles. - Explain the reasons why the domain of beliefs about cancer (strong predictor) is associated with medication adherence. - Gather strategies to support the understanding of cancer and OAMs for future interventions |
| Coherence | 2. Many patients have trouble learning about their medical condition. What could hinder your ability to learn about cancer? 3. How well prepared were you with the information you received from healthcare providers about the side effects of cancer medication?    - How does the information influence whether you take your cancer medication? 4. What do you wish you had known prior to starting your cancer medication? |  |
| Risk of recurrence | 5. What might be impacting the risk of recurrence (for cancer to come back)? 6. How does this thought influence whether you take your cancer medication? |  |
| Emotional representations | 7. What emotions do you feel about having cancer? |  |
| Cause | 8. What do you think causes cancer?    - Where did you learn about the causes of cancer? |  |
| Treatment consequences | 9. What do you see as the main effects of taking your cancer medication?    - How have the side effects of cancer medication affected your everyday life?    - How have the side effects affected how you think about your cancer medication? |  |
| Treatment control | 10. In what way does your cancer medication help you to control your cancer?     - Tell me why you (do or don’t) believe taking your cancer medication can help?     - In what way do you think taking your cancer medication helps you prevent the risk of having a recurrence?     - What could help you feel that taking your cancer medication can help? |  |
| Personal control | 11. What might be things you can do personally to control cancer (or prevent the risk of recurrence)?     - How do (the things you can do personally/personal tasks) affect whether you take your cancer medicine?     - What would you need and feel is helpful in managing cancer? |  |
| ***Section C: Beliefs about Oral Anticancer Medications (OAMs)*** | | |
| **Domains** | **Example interview questions** | **Rationale for questions** |
| Necessity beliefs | 12. Tell me why you (don’t) believe taking OAM is necessary.     - How does this belief that your cancer medication is necessary influence whether you take the medication? 13. What could other people do to help you feel that taking cancer medication is necessary? | - Understand the significant group differences in medication beliefs among three health belief subgroups/profiles. - Explain the reasons why necessity beliefs and concern beliefs (strong predictors) are associated with medication adherence. - Gather strategies to support necessity beliefs and reduce concerns for future interventions |
| Concern beliefs | 14. Tell me why you are (not) worried about your cancer medication.     - What concerns do you have about your cancer medication?     - How do your concerns influence whether you take your cancer medication? 15. What could other people do to help address your concerns about cancer medication? |  |
| ***Section D: Medication Adherence and Demographics*** | | |
| **Domains** | **Example interview questions** | **Rationale for questions** |
| Barriers to OAM use | 16. Describe any barriers that you face in taking your cancer medication     - Symptoms, side effects, transportation (if going to clinic), cost, availability, clinical decision made by HCPs, drug-drug interactions, resistance, understanding the treatment regimen, etc.) 17. What could other people do to help you address your barriers to taking your cancer medication? | - Identify barriers to adhering to OAMs among three health belief subgroups. - Gather strategies to address the barriers and support adherence for future interventions |
| Comorbidity | 18. What other chronic disease/illness do you have other than cancer?     - What is your experience managing other diseases when you have to take your cancer medicines? | - Strong predictor of medication adherence |
| Health literacy | 19. Many patients have trouble learning about their medical condition. What could hinder your ability to learn about cancer? 20. What helps you understand the information about cancer stage?     - How did they deliver the information that help you understand?     - What could be done to help you better understand the information? 21. What helps you understand the information about the cancer medications you are currently taking? (For example, the type of treatment, why you need to take this medicine, and potential medication side effects of cancer medicines)     - How did they deliver the information that helps you understand?     - What could be done to help you better understand the information? 22. What helps you learn about the support you can receive from other healthcare team members? (For example, understanding their roles in helping you recover or returning to normal life)     - What could be done to help you better understand the information? 23. What other information would you like to learn more about cancer? | - Strong predictor of medication adherence |
